# Delays in childhood immunization in a conflict area: a study from Sierra Leone during civil war

**DOI:** 10.1186/1752-1505-1-14

**Published:** 2007-12-09

**Authors:** Charles Senessie, George N Gage, Erik von Elm

**Affiliations:** 1 Department of Community Health, College of Medicine and Allied Health Science, University of Sierra Leone, Freetown, Sierra Leone, Africa; 2 Afro-European Medical and Research Network (AEMRN), Bern, Switzerland; 3 Division of International and Environmental Health, Institute of Social and Preventive Medicine (ISPM), University of Bern, Bern, Switzerland; 4 Department of Medical Biometry and Statistics, University Medical Centre, Freiburg, Germany

## Abstract

**Background:**

Sierra Leone has undergone a decade of civil war from 1991 to 2001. From this period few data on immunization coverage are available, and conflict-related delays in immunization according to the Expanded Programme on Immunization (EPI) schedule have not been investigated. We aimed to study delays in childhood immunization in the context of civil war in a Sierra Leonean community.

**Methods:**

We conducted an immunization survey in Kissy Mess-Mess in the Greater Freetown area in 1998/99 using a two-stage sampling method. Based on immunization cards and verbal history we collected data on immunization for tuberculosis, diphtheria, tetanus, pertussis, polio, and measles by age group (0–8/9–11/12–23/24–35 months). We studied differences between age groups and explored temporal associations with war-related hostilities taking place in the community.

**Results:**

We included 286 children who received 1690 vaccine doses; card retention was 87%. In 243 children (85%, 95% confidence interval (CI): 80–89%) immunization was up-to-date. In 161 of these children (56%, 95%CI: 50–62%) full age-appropriate immunization was achieved; in 82 (29%, 95%CI: 24–34%) immunization was not appropriate for age. In the remaining 43 children immunization was partial in 37 (13%, 95%CI: 9–17) and absent in 6 (2%, 95%CI: 1–5). Immunization status varied across age groups. In children aged 9–11 months the proportion with age-inappropriate (delayed) immunization was higher than in other age groups suggesting an association with war-related hostilities in the community.

**Conclusion:**

Only about half of children under three years received full age-appropriate immunization. In children born during a period of increased hostilities, immunization was mostly inappropriate for age, but recommended immunizations were not completely abandoned. Missing or delayed immunization represents an additional threat to the health of children living in conflict areas.

## Background

Sierra Leone has undergone a decade of civil war from 1991 to 2001 characterised by military action targeted against civilians, including many children [1]. A government report has documented more than 40.000 violations of human rights [2]. However, the actual number of victims is estimated at about 50.000 deaths and more than a million internally displaced people [1]. The direct consequences of war have been aggravated by the destruction of infrastructure and the loss of skilled personnel in all sectors including health care [3]. Eight years after the Lomé Peace Agreement, Sierra Leone is today amongst the countries with the greatest shortfall in development [4]. In 2000, about 17% of Sierra Leone's population of 4.5 million were under five years old [5].

The adapted Expanded Programme on Immunization (EPI) had been implemented in Sierra Leone since 1974 and covered six major childhood diseases (Table 1). By immunization campaigns on national immunization days and through mobile outreach teams the immunization coverage for these diseases could be increased significantly during the pre-war period. For instance, coverage for DTP3 increased from 13% in 1980 and 22% in 1988 to 83% in 1990 [6]. By 1990, at least 75% of children aged 12 to 23 months were found to be fully immunized for each of EPI's six target diseases in a national survey [7]. In a 1990 survey in the Greater Freetown area, 89.4% of children aged five years or less were immunized against BCG, 77.3% against DPT, 75.8% against polio, and 61.8% against measles, respectively [8]. At this time, the infant mortality rate decreased from 162.3 per 1000 live births in 1985–87 to 69.9 per 1000 live births in 1988–89 [8]. From the ensuing period of civil war reliable data on immunization coverage were no longer available and time trends could not be estimated. At the end of the civil war in 2000, the immunization coverage for all diseases targeted by EPI was similar to or below the levels of 1988. As a consequence of the efforts by the donor agencies, in particular the United Nations Children Educational Fund (UNICEF) and the Global Alliance for Vaccinations and Immunization (GAVI), it could be raised gradually during the post-war period [3,6]. However, 282 of 1000 Sierra Leonean children still died before the age of five years in 2005 [5]. Enhancing the immunization coverage remains the primary goal of these efforts. Differences between age-appropriate and up-to-date status (i.e. immunization delays) have not been investigated neither before nor during the war.

**Table 1:** Schedule for childhood immunization in Sierra Leone based on Expanded Programme on Immunization (EPI)

**Time point**	**Disease**	**Vaccination**
Birth	Tuberculosis	BCG
6 weeks	Diphtheria/Tetanus/Pertussis + Polio	DTP-1 + OPV-1
10 weeks	Diphtheria/Tetanus/Pertussis + Polio	DTP-2 + OPV-2
14 weeks	Diphtheria/Tetanus/Pertussis + Polio	DTP-3 + OPV-3
9 months	Measles	Measles
15 months	Diphtheria/Tetanus/Pertussis	DTP booster
18 months	Measles	1^st ^measles booster
24 months	Measles	2^nd ^measles booster*

* not practised in Sierra Leone during study period

It is well known that the direct and indirect consequences of conflicts amplify health risks due to communicable diseases [9]. Populations of conflict areas are often faced with the re-emergence of diseases that had been under control or even eradicated locally [10,11]. For instance, in 2004 an outbreak of Lassa fever in the Kenema district in Sierra Leone was due to the long-term deterioration of infection control practices in the local hospital [12]. Children are particularly vulnerable to infectious diseases if their immunity is compromised by malnutrition [9]. In humanitarian interventions in conflict areas, timeliness of immunization against vaccine-preventable diseases is a priority because any delays put children at additional risks of infection [13-16].

The aim of this study was to estimate childhood immunization coverage in a Sierra Leonean community during the civil war period. Specific objectives of the present analysis were to determine the immunization status in different age groups of children aged three years or less and to explore potential temporal associations between immunization status by age group and war-related hostilities.

## Methods

### Setting

**Figure 1:**
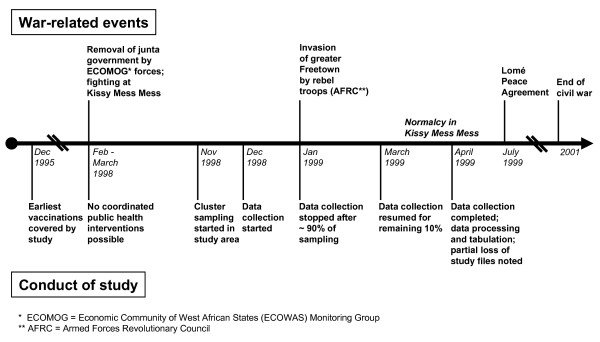
Time relationship between war-related events and conduct of study.

Kissy Mess-Mess is a community in the Eastern part of Greater Freetown. The community was chosen for two reasons: First, its infrastructures in transport and communication were deemed sufficient and safe enough for fieldwork due to the proximity of the capital city. Second, it comprised both urban and rural residential areas and had been affected by hostilities already in the past (i.e. during 1998). The population was about 200.000 in 1999, including three large camps with mostly internally displaced people. Health care available to the resident population was based on primary health care services provided by a peripheral health unit (PHU) of the Maternal and Child Health Division of the Sierra Leone Ministry of Health, a maternity hospital of the Marie Stopes Society and a private clinic. About half of immunizations were delivered by the PHU. Other organisations that were active in immunization campaigns in the community before the onset of hostilities included UNICEF, the Parliamentary Action Group on Child Survival, the Islamic Action group, the Christian Health Association, and Médicins Sans Frontières (MSF). However, with increasing insecurity the foreign organisations were forced to stop their activities and leave the country. From early 1998 onwards, Greater Freetown was temporarily under siege and any coordinated public health interventions became almost impossible. Later, Kissy Mess-Mess was repeatedly assaulted by rebel troops. Figure 1 shows the temporal relationship between war-related events and the conduct of our study. Briefly, the data collection lasted from December 1998 to March 1999 (Figure 1). Unfortunately, the insecurity also impacted on our study: The study locations in the Eastern part of the city were assaulted by rebel troops in early 1999. The individual data entry forms of our study were destroyed. Consequently, all analyses presented here are based on the aggregated data that had been secured before.

### Study population and sampling

Children were eligible if they were aged three years or less at time of interview and lived in Kissy Mess-Mess. It was decided to include children below age of 12 months in order to be able to collect data on tetanus toxoid coverage at the same time (data not shown) and to obtain data on the most recent immunizations. We defined the following age groups: (I) 0–8 months, (II) 9–11 months, (III) 12–23 months, and (IV) 24–35 months. We assumed that immunization status in these age groups reflects the availability of immunization services to children at the time when they were eligible for an immunization.

We used an adapted two-stage sampling method with mutually exclusive strata and random sampling of households within strata [17]. On a map the community was arbitrarily divided into 30 strata with approximately similar number of households (defined as a "compound" i.e. a circumscribed living place). We excluded children from refugee and internally displaced camps because their immunizations were carried out in the camps and health care delivery there differed from residential areas. Our interview teams comprised community health officers, nurses, and medical students experienced in survey data collection. In each stratum, teams sampled every third household starting at a randomly chosen location. Heads of households were asked for participation; if they consented, the household's youngest child was included. We aimed to obtain a minimum sample size of 210 (i.e. 30 × 7) as recommended for rapid immunization surveys, but stopped sampling only after 8 to 10 children per stratum to allow for missing data [17].

### Data collection

Interviewers asked mothers or guardians to bring children on site, and to show immunizations cards. Generally, the so-called "under five cards" are issued at birth and dates of vaccinations are noted subsequently. In most households the cards are kept in a hard plastic bag that is delivered at the same time. The families often used these bags to store valuables, money and other documents, which helped to achieve high card retention rates. If cards were unavailable, we took verbal histories and also checked for bracelets ("bangles") from immunization campaigns at the wrists of infants of one year or less. If possible, we used records of the Births and Deaths Register and the local health centre to verify verbal histories. If information was unclear, the child was excluded. All children were examined for typical BCG scars. We compared BCG coverage evidenced by scar and immunization card to assess the reliability of collected data.

The WHO "Infant immunization cluster form" was used to collect data on the number of children, dates of birth, type and dates of each vaccine dose [18,19]. If there were any departures from the regular immunization schedule, mother or guardians were asked for reasons using an open-ended question. Given the threatening circumstances of civil war, the interviewers did not ask specifically for war-related reasons. Answers were categorised according to the WHO "Reasons for immunization failure cluster form" [18].

### Definitions

Each child's immunization record was checked against the EPI immunization schedule including booster doses for DTP at 15 months and for measles at 18 months (Table 1). To account for age-appropriateness of given immunizations, we used the WHO standard definition for up-to-date immunization status [18,20], but subdivided it into *full age-appropriate immunization *and *age-inappropriate immunization*. Consequently, we used the following four categories: *full age-appropriate immunization*, if all vaccinations recommended in the EPI schedule were given in time according to the child's age on the day of interview; *age-inappropriate immunization*, if all recommended vaccinations were given, but one or more were given later (= 1 day) than the scheduled date; *partial immunization*, if at least one recommended vaccine dose was not given; and *not immunized *if none of the recommended vaccinations was carried out. Our main outcome was the proportion of children with *full age-appropriate immunization *status.

Card retention rate was defined as the proportion of children whose immunization cards were available. We also calculated two drop-out rates to study the utilization of the immunization system. Drop-out rate for DPT-1-to-3 period was defined as the proportion of children with DPT-1 dose but without subsequent DPT-3 dose. Drop-out rate for BCG-to-measles period was defined as the proportion of children with BCG vaccination but without subsequent measles vaccination.

### Statistical analysis

We used descriptive statistics and calculated binomial 95% confidence intervals (95%CI) for proportions indicating immunization status for age. Although stratified random sampling may increase precision as compared to simple random sampling, we did not account for a potential design effect <1 in order to yield more conservative estimates [18]. We tested whether the distribution of immunization status for age differed between age groups using the χ^2 ^test. Microsoft Excel was used for data tabulation, and Stata 8.2 for statistical analyses. The study protocol was examined by members of the research ethics committee of the Sierra Leone Medical & Dental Council.

## Results

### Participants

In total, 286 children aged three years or less from all 30 pre-defined clusters were included. Forty-six children (16%) were aged 0–8 months, 58 (20%) aged 9–11 months, 83 (29%) aged 12–23 months, and 99 (35%) aged 24–35 months. Few households refused participation; their exact number was not recorded.

Of a total of 1690 vaccine doses administered; 916 (54%) were given by the primary health unit, 419 (25%) by the maternity hospital or the private clinic, 224 (13%) by outreach teams, and 131 (8%) by a government hospital. Overall card retention was 87%. In the age groups I to IV it was 85%, 86%, 89%, and 87%, respectively. For 37 children (13%) information was not based on immunization cards. In two clusters data were mostly obtained by verbal history because houses in these areas were burnt down by rebel troops in January 1999.

### Immunization status

Overall, 85% (95%CI: 80–89%) of children had up-to-date immunization according to the WHO definition (Table 2). This proportion is composed of 56% (95%CI: 50–62%) of children with age-appropriate immunization, and 29% (95%CI: 24–34%) with age-inappropriate immunization (Table 2). In age groups I, III, and IV the proportion of age-appropriately immunized children ranged from 57% to 69%. However, in children aged 9–11 months (age group II) only 28% (95%CI: 17–41%) of children were age-appropriately immunized and 52% (95%CI: 38–65%) were age-inappropriately immunized (Table 2). There was strong statistical evidence that immunization status differed according to age group (χ^2 ^= 31.3 df = 9, p < 0.0001).

**Table 2:** Immunization status of children as compared to the Expanded Programme on Immunization (EPI) schedule

Age group (months)	I (0 – 8)	II (9 – 11)	III (12 – 23)	IV (24 – 35)	Total
Total number	46	58	83	99	286
%	100	100	100	100	100
Up-to-date immunization	40	46	74	83	243
% (95%CI)	87 (74–95)	79 (67–89)	89 (80–95)	84 (75–90)	85 (80–89)
*Full age-appropriate immunization*	26	16	51	68	161
*% (95%CI)*	57 (41–71)	28 (17–41)	61 (50–72)	69 (59–78)	56 (50–62)
*Age-inappropriate immunization*	14	30	23	15	82
*% (95%CI)*	30 (18–46)	52 (38–65)	27 (18–39)	15 (9–24)	29 (24–34)
Partial immunization	5	10	8	14	37
% (95%CI)	11 (4–24)	17 (9–29)	10 (4–18)	14 (8–23)	13 (9–17)
No immunization	1	2	1	2	6
% (95%CI)	2 (0–12)	3 (0–12)	1 (0–7)	2 (0–7)	2 (1–5)

95%CI = binomial 95% confidence interval

The coverage for single vaccine doses (irrespective of time of vaccination) is given in Table 3. Overall, coverage for individual vaccine doses declined across age groups from older children (age group IV) to younger children (age group I) (Table 3). Both OPV-3 and DTP-3 coverage were only 54% in children aged 0–8 months; and measles coverage was only 28% in children aged 9–11 months.

**Table 3:** Coverage for individual vaccine doses

Age group (months)	I (0 – 8)	II (9 – 11)	III (12 – 23)	IV (24 – 35)	Total
	Number (%)	Number (%)	Number (%)	Number (%)	
Total in group	46 (100)	58 (100)	83 (100)	99 (100)	286 (100)
BCG	39 (85)	48 (83)	73 (88)	91 (92)	251 (88)
OPV-1	38 (83)	49 (85)	71 (86)	85 (86)	243 (85)
OPV-2	34 (74)	45 (78)	64 (77)	77 (78)	220 (77)
OPV-3	25 (54)	37 (64)	57 (69)	71 (72)	190 (66)
DTP-1	38 (83)	49 (84)	70 (84)	84 (85)	241 (84)
DTP-2	34 (74)	45 (78)	63 (76)	76 (77)	218 (76)
DTP-3	25 (54)	37 (64)	58 (70)	70 (71)	190 (66)
Measles	-	16 (28)	55 (66)	68 (69)	139 (58)*
DTP Booster	-	-	10 (12)	8 (8)	18 (10)**
Measles Booster	-	-	14 (17)	11 (11)	25 (14)**

* Proportion based on 240 children eligible for vaccination.

** Proportion based on 182 children eligible for vaccination.

Based on immunization cards, BCG vaccination was performed in 251 (88%) of all children (Table 3). On examination, BCG scar was present in 249 (87%) children. In age groups I-IV, BCG vaccination was documented in 39, 48, 73, and 91 children, respectively. A BCG scar was present in 37, 43, 76, and 93 children, respectively. The drop-out rate for DPT-1-to-3 period was 21%. In age groups I to IV it was 34%, 25%, 21%, and 20%, respectively. The drop-out rate for BCG-to-measles period was 44%. In age groups II to IV, it was 63%, 28%, and 27%, respectively.

### Temporal association with war-related events

Mothers or guardians of 125 (44%) children gave reasons why immunizations were not carried out. Most frequent reasons include "Mother too busy" (n = 25, 20%), "Fear of side reactions" (n = 10, 8%), and "Postponed until another time" (n = 10, 8%). War-related events were not mentioned. Possibly, underlying reasons such as insecurity were not openly expressed to interviewers and secondary factors given instead.

Most children aged 24–35 months at the time of interview were born between December 1995 and December 1996 and received most EPI immunizations during a period of relative security in Kissy Mess-Mess. A majority of these children were age-appropriately immunized (Table 2). In contrast, children aged 9–11 months at the time of interview were mostly born in the first three months of 1998 when the community was under siege. Only 28% of these children were age-appropriately, and in 52% immunization was age-inappropriate (Table 2).

## Discussion

We studied immunization of children in a Sierra Leonean community during the civil war. In children aged three years or less the proportion of full age-appropriate EPI immunization was 56% (95%CI: 50–62%), and of age-inappropriate immunization 29% (95%CI: 24–34%). The immunization status and delays in immunization varied across age groups, and temporal associations with war-related events in the community could be identified.

### Limitations and strengths

In general, systematically collected health data of populations living in conflict areas are scarce [14]. Consequently, little is known about immunization coverage and health status in areas that are too insecure to conduct population-based research [21]. In our study, loss of confidence in traumatized residents limited the interviewers in what information they could ask for, as described for similar settings [22]. In particular, war-related reasons for departures from the immunization schedule could not be addressed directly.

As a second limitation, our results are likely not representative for the entire country, in particular rural areas. However, there were no other immunization studies in the country during that time, at least to our knowledge. Also, the sampled households may not be representative of the community. Eligible children may have died or been displaced before. If the immunization of these children was more often age-inappropriate or partial this might have led to an overestimation of age-appropriate immunization.

Third, when analysing the immunization coverage in different age groups, we assumed that it reflects the immunization practice during the three years before data collection. However, the immunization status of enrolled children may have been influenced by other factors. For instance, problems with vaccine supply and cold chain could have been unrelated to war. However, we are not aware of such problems in the community at this time. Of note, national mass immunization campaigns were no longer carried out in Sierra Leone after the outbreak of the civil war in 1991 and were resumed only towards the end of the war in the context of humanitarian cease-fires [23].

We focused on *age-appropriate *immunization because the timeliness of vaccinations is most important in children who are at increased risk of vaccine-preventable diseases, as is the case in conflict areas [24]. However, when estimating age-inappropriate immunization, we could not record the actual length of delays. If a missed immunization had been carried out soon after the scheduled date it would have been counted as delayed. Similarly, children with partial immunization may have missed a scheduled vaccination only for a few days. A follow-up of surveyed children could have accounted for this, but was not feasible. These circumstances need to be taken into account when interpreting the results of this study.

### Comparison with other studies

Our estimates for immunization coverage likely differ from previous studies conducted in Sierra Leone due to different definitions. For instance, maximum age in a previous study on immunization in Sierra Leone was five years [8]. Also, coverage studies usually do not collect data from children below age of 12 months. We compared each child's immunization status at the time of interview with the EPI schedule to estimate age-appropriate immunization, while other studies reported on up-to-date status only [8,25]. In a review of 48 interventional immunization studies, a majority estimated only up-to-date but not age-appropriate immunization [20]. The current literature on age-appropriate immunization is limited. Of ten such studies identified in a recent overview, seven were conducted in the USA, two in Australia and one in Sweden [26]. The proportion of children with age-appropriate immunization ranged from 6% to 75% and was associated with factors such as ethnicity, residence, poverty, or vaccine type [26].

The up-to-date immunization coverage across all included age groups was 85% in our study. This high proportion of immunized children includes about 29% of all children who did not receive vaccine doses *on time*. The magnitude of immunization delays has been investigated in other settings, and statistical methods were proposed to account for such delays [24,27,28]. In a coverage study conducted in the USA in 1991/92 age-appropriate immunization was at least 37 percent points lower than up-to-date immunization for each of DTP, OPV, and MMR [27]. In a study conducted in Argentina in 2002, 38% of children had delayed DTP4 immunization and 36% delayed MCV immunization [28]. Considerable delays in all three doses of a pentavalent vaccine against DTP, Haemophilus influenzae type b, and Hepatitis B were found in a coverage study in Kenya in 2002 [29].

Statistical evidence supported the hypothesis that children aged 9–11 months were less often fully immunized for age than children of other age groups. Their immunization was mostly delayed. Vaccinations missed during the first 3 to 4 months of life appeared to have been made up for as soon as insecurity diminished. This may indicate that, despite ongoing hostilities, health care providers as well as mothers may have continued to give childhood immunization high priority. We were unable to determine specific reasons for this achievement. It would be worthwhile to investigate which importance mothers or other caregivers in conflict areas attribute to childhood immunization, for instance by using qualitative research methods. Of note, immunization campaigns were among the few reasons for which cease-fires have been negotiated in several countries affected by armed conflicts, including Sierra Leone [23].

During the civil war in Sierra Leone, rural areas were affected more seriously and over a prolonged period of time. In Angola, a considerable rural-urban difference in immunization coverage was shown in a study on child health and civil war: the drop in immunization coverage was more pronounced for children in rural than in urban areas [13]. Likewise in Angola and other conflict areas, the breakdown of public health services was even more pronounced in the rural areas of Sierra Leone and aggravated by increased expenditure on military instead of health [14]. Our data from a semi-rural community on the outskirts of the capital city may not reflect this situation entirely. In choosing this setting we aimed to approximate the rural situation as closely as possible under given circumstances.

### Importance of study results

Direct and indirect consequences of civil war are known to amplify pre-existing health risks caused by malnutrition and infectious diseases [13,14]. During humanitarian interventions in conflict areas timely immunization is considered important [15]. Any delay puts children at an additional preventable risk of death. Also, delayed immunization may indicate substantial inequality in the access to other public health interventions and to health care in general [24]. It is therefore important to detect and document delays in immunization (and the reasons thereof) in conflict areas where health services may still be available to some extent. Consequently, we propose that coverage studies use both age-appropriate immunization and up-to-date status as an indicator in paediatric populations that are at high risk of vaccine-preventable diseases.

## Conclusion

We found a low proportion of children with full age-appropriate immunization in a Sierra Leonean community exposed to war-related hostilities while up-to-date immunization was maintained. This indicates that many of the missed vaccinations were caught up for later. Lower levels of full age-appropriate immunization were found in children in whom the regular EPI schedule could likely not be followed due to specific war-related events impacting on the community. Such delays in immunization represent an additional threat to children living in conflict areas. They can only be investigated if studies use age-appropriate immunization in addition to up-to-date immunization as an indicator.

## Competing interests

The author(s) declare that they have no competing interests.

## Authors' contributions

CS participated in the design of the study, supervised and coordinated the field work, collected and processed the data, drafted the manuscript, and is study guarantor. GNG planned the study, participated in its design, and supervised the study. EvE analysed and interpreted the data, and drafted and revised the manuscript. All authors approved the final manuscript.
